# Prevention and control of childhood asthma and allergy in the EU from the public health point of view: Polish Presidency of the European Union

**DOI:** 10.1111/j.1398-9995.2012.02822.x

**Published:** 2012-04-28

**Authors:** B Samoliński, A Fronczak, P Kuna, C A Akdis, J M Anto, A Z Bialoszewski, P G Burney, A Bush, A Czupryniak, R Dahl, B Flood, G Galea, M Jutel, M L Kowalski, S Palkonen, N Papadopoulos, F Raciborski, D Sienkiewicz, A Tomaszewska, E Mutius, D Willman, A Włodarczyk, O Yusuf, T Zuberbier, J Bousquet, Bodo Niggemann

**Affiliations:** 1 Department of Prevention of Envinronmental Hazards and Allergology, Medical University of WarsawWarsaw, Poland; 2 Ministry of HealthWarsaw, Poland; 3 National programs against asthma and the Polish national asthma program – Polasthma, Medical University of LodzMedical University of Lodz, Łódź, Poland; 4 European Academy of Allergy Clinical Immunology (EAACI), Swiss Institute of Allergy and Asthma Research (SIAF), Christine Kühne-Center for Allergy Research and EducationDavos, Switzerland; 5 Centre for Research in Environmental Epidemiology (CREAL)Barcelona, Spain; 6 Imperial CollegeLondon, UK; 7 Imperial College and Royal Brompton HospitalLondon, UK; 8 Department of Respiratory Diseases, Aarhus University HospitalAarhus, Denmark; 9 European Federation of Allergy and Airways Diseases Patients' Associations (EFA)Brussels, Belgium; 10 WHO EuropeCopenhagen, Denmark; 11 Department of Clinical Immunology, Wroclaw Medical UniversityWroclaw, Poland; 12 Department of Immunology, Rheumatology and Allergy, Medical University of ŁódźŁódź, Poland; 13 Department of Allergy, European Academy of Allergy Clinical Immunology (EAACI), 2nd Pediatric Clinic, University of AthensAthens, Greece; 14 European Public Health AllianceBrussels, Belgium; 15 University Children's HospitalMunich, Germany; 16 Silvermedia, Sp. z o. o. Sp. k.Krakow, Poland; 17 The Allergy & Asthma InstituteIslamabad, Pakistan; 18 Department of Dermatology and AllergyCharité, Berlin; 19 University Montpellier-1 and Inserm CSEP 1018, WHO Collaborating Center for Asthma and RhinitisMontpellier, France

**Keywords:** asthma, allergy, rhinitis, specific immunotherapy, risk factors, public health

## Abstract

The leading priority for the Polish Presidency of the Council of the European Union was to reduce health inequalities across European societies, and, within its framework, prevention and control of respiratory diseases in children. This very important paper contain proposal of international cooperation on the prevention, early detection and monitoring of asthma and allergic diseases in childhood which will be undertaken by the EU member countries as a result of EU conclusion developed during the Polish Presidency of the Council of the European Union. This will result in collaboration in the field of chronic diseases, particularly respiratory diseases, together with the activity of the network of national institutions and NGOs in this area. Paper also contains extensive analysis of the socio-economic, political, epidemiological, technological and medical factors affecting the prevention and control of childhood asthma and allergy presented during Experts presidential conference organized in Warsaw-Ossa 21–22 September 2011.

## Forewords

Mrs Ewa Kopacz/Andrzej Włodarczyk, *Minister of Health*, Pr Marek Krawczyk, *Rector of Medical University of Warsaw*, Pr Bolesław Samoliński, *Chairman of the Sub-Committee on Priorities of Polish Ministry of Health*.

The leading priority for the Polish Presidency of the Council of the European Union is to reduce health inequalities across European societies and, within its framework, prevention and control of respiratory diseases in children. The conference will draw attention to allergy and asthma being the most common developmental age diseases and of great impact on health in later stages of life. The title of the conference obliges to take dynamic action to eliminate most urgently inequalities in health, particularly in such an important subject matter as respiratory diseases. What counts is a concrete action, considering a joint action in the field of chronic diseases, particularly respiratory diseases, together with the activity of the network of institutions collaborating in this area. Closing remarks to the debate is aiming to the preparation of the proposal of the EU conclusion on early detection, prevention and monitoring of noninflammatory respiratory diseases, with a particular consideration of the population of children and young people. This is a crucial aim and an extremely important conference – one of the most important in the course of the Polish Presidency of the Council of the European Union. The summary of this conference was used to draft the Conclusions of the Council of the European Union on prevention, early diagnosis and treatment of chronic respiratory diseases in children 1.

## Rationale: scientific evidence of the priority

Despite the recent development, the understanding of allergic diseases is insufficient, patient care is suboptimal and public awareness is needed. Novel knowledge on the origin and mechanisms of the allergic diseases should be generated to understand how an epidemic increase in allergic diseases took place without any change in hereditary genetic predisposition 2. Global networking among all stakeholders is essential to develop transdisciplinary strategies towards allergy prevention, better patient care and new therapeutic options.

### Reasons for the priority

Chronic respiratory diseases put a high burden on public health in Europe. Around 25% of the population in Europe (130 million) suffers from allergic rhinitis, 30 million from asthma. Rhinitis in children increases the risk of asthma, and asthma is a major risk factor for chronic obstructive pulmonary diseases (COPD) #b^3^b^4^. Allergy and asthma often start in children, but can persist throughout life. The course of the disease is affected by several factors, which, until now, have been listed as priorities during individual EU presidency periods: for example, smoking, environmental pollution and socioeconomic determinants. Therefore, it seems justifiable to develop a new vision to integrate all health problems in Europe. The issues of chronic diseases appeared in the plans for the Belgian presidency of the EU 5 are included in the Polish Presidency programme. To balance the differences in providing full and efficient healthcare systems, all stakeholders should fight with the growing epidemics and improve the quality of life of those already affected by such diseases.

### Epidemiology of asthma and allergy shows inequalities in the EU

Wide variations in the prevalence of allergy exist across Europe both in children [ISAAC 6] and in adults [ECRHS 7]. Symptoms of allergic disease have been rising globally at least to the mid-1990s. Whatever happens in new sensitizations in children, because of very high prevalence in young ages, the ‘epidemic’ of allergic diseases will be growing in the ageing population for several decades to come.

### Socio-economic determinants in allergy and asthma

Asthma is more common and more severe in populations with a low socio-economic status although allergic diseases may be commoner in families of a high socio-economic status 8. Many risk factors are involved in the social determinants in asthma including environmental factors (maternal smoking, air pollution, housing conditions including allergen levels), diet, sedentary life style and stress. These risk factors may act at individual and community-area levels. In addition, susceptibility factors like birth weight, prematurity and obesity are associated with impaired lung function contributing to the increasing risk of asthma among underserved populations.

### Risk factors of allergy and asthma in early life

In some rural areas, allergic diseases occur rarely, whereas in urban environments they are more frequent. Environmental factors determining allergy onset occur before the clinical manifestation of disease, that is during pregnancy and infancy, and can impact maturation and development of the immune system 9. Protective influences will result in tolerance towards food and inhalant allergens. In turn, risk factors will impact the development of an individual's capacity to mount IgE antibodies to otherwise harmless allergens and to manifest with clinical disease. Early contact to environments rich in microbial exposures in farms and traditional rural settings may be protective. In contrast, second-hand smoke and urban traffic-related pollution have effects, particularly on asthma.

### Childhood influences on later lung health

*In utero* and early life environments are important determinants of adult asthma or COPD 10. Adverse effects on the foetus can result from the behaviour of previous generations. In foetal life, second-hand smoke is the most important risk factor. Other adverse factors are environmental pollution, diet and medications (antibiotics and paracetamol for example). Cohorts have delineated the changes in lung function over time in children with wheezing and/or persistent asthma 11. Early sensitization to aeroallergens is an important predictor of asthma. Lung function tracks through school age into adult life and there is no catch-up growth, and damage made in the preschool years is permanent. Continued exposure to second-hand smoke and environmental pollution further impairs lung growth 12. The effects of lung injury in the antenatal period and preschool years are long-lasting and irreversible. Adult lung health can only be achieved by sustained targeting of preventive measures in these critical early years. This is where public health measures can have the greatest impact.

### Prevention of chronic respiratory diseases in childhood and its impact later in life

Asthma starts early in life. To be able to predict the persistence of the disease, we need to better understand its mechanisms of progression. Furthermore, measures to prevent such progression will have a major impact in the quality of life of Europeans and public health outcomes. Many cases of asthma start with a viral infection. ‘PreDicta’, an FP7 EU-funded project, is currently scrutinizing the mechanisms of such progression. Allergen avoidance is difficult to achieve, but suggests some effect when performed thoroughly 13. Prevention by antiviral treatment has been shown in principle by an antibody to respiratory syncytial virus; however, a vaccine against the common cold virus remains elusive. Finally, second-hand smoke exposure should be avoided. The Finnish Programme on allergy is a national example to be followed 14.

### e-Health in allergy: Internet system of early allergy and asthma diagnosis, the Polish experience

In Poland, allergic symptoms are found in 40% of 6- to 7-year-old children 15. Late recognition of these diseases is a serious problem, and many patients consult several years after the onset of symptoms 15. The current system of basic health care cannot adequately provide early diagnosis of allergic diseases. Moreover, not all patients with positive skin tests present allergic symptoms. Early recognition of asthma and allergy within the scope of e-Health is therefore required. The rapid development of technology in health care combined with improved access to the Internet has opened new perspectives within this area. On the basis of the available data, an Internet system has been created for the early detection of asthma and allergies. Advanced algorithms based on the ECAP data 15 are able to evaluate the risk of allergy disease occurrence and personalize suggestions for further procedure.

### National programmes against asthma and the Polish national asthma programme – POLASTMA

National Programme of Early Diagnosis and Asthma Therapy – POLASTMA – is an all-Polish pro-health action 16. It is based on a widely led education in medical society and general population, which aims to facilitate the patients' access to medical care and to improve its quality. The Programme is a reaction of medical society to increasing problems concerning asthma in Poland. Examples from Finland, Poland and Brazil show that asthma burden can be reduced using strategies in different societal, economical and healthcare environments. Regardless of the healthcare system and its coverage, reduction in asthma burden is possible through cost-saving national or regional asthma plans.

### Specific immunotherapy – modern prevention and prophylaxis in allergy and asthma

Allergen immunotherapy is a hallmark of allergy treatment as shown by meta-analyses #b^17^b^18^, and evidence-based guidelines show that it is effective in respiratory allergies 13. It is the only treatment affecting the natural course of the disease. Allergen immunotherapy is effective in alleviating allergy symptoms to a similar (or even larger) extent as pharmacological treatments 19, although clinical trials should be considered differently 20. Persistence of clinical benefits is found after immunotherapy has been discontinued. Immunotherapy has also been demonstrated to prevent the progression of allergic diseases and the development of new sensitizations so it could be treated as secondary prevention method. The ultimate goal of specific immunotherapy is to restore the immune tolerance as it is active in the healthy individuals 21.

### The patient's perspective

Patient perspective in allergy can be summarized in one sentence; the priorities are quality of life – normal life despite the disease, cure and prevention. A specific issue is the role of environmental issues, including air, food, chemicals in our living environment and products. Patient perspective as represented by patient organizations arises from the collective experience of patients living with allergy, their parents and partners. Another perspective is the individual perspective of given patient (including children) arising from their individual experience of living with the disease in their specific circumstances, challenges and goals. Patient associations see the management of allergy as a comprehensive approach that takes into account care and environment. Many policy areas must play a role and encourage comprehensive multidisciplinary national programmes on allergy and involve all stakeholders. Patient perspective should be incorporated at all levels of prevention and care. Patient perspective is powerful and a practical tool to complement medical and other scientific expertise.

### Importance of allergy and asthma in the chronic disease epidemic and related costs

Asthma and allergy burden is substantial in Europe and in most countries in the world 4. Because of the large amount of people affected in their school and productive age, the economic burden of lost productivity and of healthcare costs attributed to asthma and allergic diseases is extremely large. Asthma is one of the most common causes for hospital admission in childhood, which can be prevented by education and optimal treatment even in underserved populations. Most but not all patients with allergic rhinitis can be controlled by optimal pharmacologic treatment. Patients with treatment resistant diseases are those with poor quality of life, school and work performance. A heavy burden is more common in asthmatics with obesity, frequent respiratory symptoms and low lung function. The trends in prevalence of allergic diseases are likely to increase their burden.

## Chronic respiratory diseases and allergy in EU health policy

## Socioeconomic determinants, inequities in health, poverty and economic consequences

One particular concern is that people with low socio-economic status bear a disproportionate burden of allergic diseases. The European Commission considers addressing respiratory diseases a key priority and addresses them from different angles. These include legislation to address tobacco consumption and ambient air quality, work on climate change, and actions under the European Environment and Health Strategy. There is also a specific policy focus on reducing health inequalities and chronic disease through the 2009 European Commission Communication on reducing health inequalities in the EU. These policies tie in with active Commission support for the current United Nations Process to address noncommunicable diseases and related socioeconomic and environmental determinants. In addition, every year the European Commission supports research and networking actions to obtain better data and identify adequate policy responses to combat respiratory disease.

## Patient's empowerment in medical decisions

The goal and rationale of patient involvement in medical decisions is patient empowerment. Empowered patient knows their disease, have the skills and motivation to take good care in their everyday life, adjust treatment and be prepared in new or potentially exacerbating situations, detect side-effects, take contact with healthcare professional when needed and adhere to treatment regime. Many tools support empowerment, shared decision making models and patient education. Patient empowerment should be included in healthcare professionals' curriculum. International guidelines in allergy and asthma (ARIA, GINA) recognize the need for patient involvement and empowerment. Another key aspect of patient involvement in medical decisions is the patient representatives' involvement in the healthcare policy and organization in practice. The members of EFA have developed tools to help in involvement in medical decisions and empowerment (http://www.efanet.org/about/members.html) and the EPF guidance to involve patients and patient organizations in EU's health-related projects and policy and a database of patient groups.

## Primary care, the cornerstone for the prevention and control of asthma and allergic diseases

Primary care physicians treat the majority of patients with respiratory diseases 22. Primary care perspectives should be fully involved in respiratory medicine and allergy to raise standards of care in individual countries and globally, through collaborative research, innovation and dissemination of best practice and education. With the development of rhinitis and asthma guidelines and the high prevalence of these diseases seen in primary care settings, it is important to investigate the knowledge, attitudes and practices of primary care physicians with regard to these guidelines.

## Allergy and Asthma in European Research Programmes

Over the last decades, because of the joined efforts of European scientists, a significant progress in the understanding of epidemiology and pathophysiology of asthma and allergic diseases has been achieved, leading to the development of new prevention strategies and implementation of new treatment modalities. Launched in 1984, research Framework Programmes (FP) of the European Union started to have significant impact on research in Europe. The ECHRS 23 was one of the earliest programme in asthma and allergy, and several research projects in this field have been funded over last two decades 24. One successful project is the network of excellence GA^2^LEN (FP6) 25, which included more than 500 scientists from 26 European countries and brought together previously fragmented European research. The allergy and asthma community has launched, within FP7 (2007–2013), several new innovative projects [e.g. FAST, Predicta, MedALL 2], which address critical gaps in our knowledge in this field and will hopefully have a significant impact on the well-being of the European society.

## Pan-European surveillance system of allergy and asthma

GA^2^LEN (FP6) 25 has standardized allergy practice and skin tests across Europe. These studies have established the basis for an allergy surveillance network. It was found that some invasive species (e.g. ragweed) were spreading in areas, where they were not expected. This surveillance work has already assisted European cities, such as Montpellier, Vienna or Berlin, to develop pro-active tactical steps in counteracting new trends in pollen allergy. This is particularly relevant to mitigate climate changes because global warming can spread species known to be allergic (e.g. cypress or birch). However, whilst the infrastructure of GA²LEN is fully in place, this surveillance project was limited within the FP6. Rapid changes in allergens are also observed in contact allergy and food. GA²LEN's existing infrastructure of more than 100 centres covering all areas of the European Union 25 offers an ideal and cost-saving platform to fill the gap to plan for the future prevalence and burden of allergy and asthma.

## Allergy and asthma in health policies of EU, focusing on childhood as a determinant of healthy ageing

The conference had an excellent timing, happening just after the UN Summit on the Prevention and Control of Non-communicable diseases such as chronic respiratory diseases (CRD), cardiovascular diseases, cancer and diabetes 26. NCDs are the biggest public health threat in the European Region 27. The 2010 Global Risk Report ranks NCDs as the second most severe threat to the global economy. Only for CRD, the total financial burden amounts to over 100 billion €. Starting in early childhood, the foundations for healthy or weakened respiratory organs are laid from a political perspective to feed into the Europe 2020 strategy's goals and healthy and active ageing 28. Multisectoral prevention, including policy change, regulation and market intervention are of the highest priority. What is needed at the EU and Member States level is the increase in investment in prevention practice and research (‘Health for Growth’ Public Health Programme and the FP8), improved physical and social environment, and promotion of lifestyle of beneficial value for ‘whole of society’ and ‘vulnerable groups’ in particular 5. The European dimension of the NCD burden needs to be fully realized, proper financial resources to public health programmes given, existing WHO resolutions, EU directives, previous relevant Council Conclusions implemented, acknowledge the important role of civil society and academia to be played in this regard.

## EU cooperation in preparing the action plan on the prevention, early detection and monitoring of asthma and allergic diseases in childhood

The conference underlined the need to find innovative ways for cost-effective prevention and management of allergy and asthma in children to:

- make healthy choices easier to reduce risk factors for the onset and progression throughout life of allergy and asthma;
- take into account socioeconomic inequalities;
- develop patient-centred policies focused on patients' needs in cooperation with the relevant stakeholders, especially patients' organizations at all levels of care including primary health care to avoid progression into chronic respiratory diseases in adulthood and later in life;
- improve knowledge and education of all stakeholders;
- develop constant monitoring and surveillance of asthma and allergy using existing networks;
- stimulate integrated research;
- create or strengthen a sustainable network of institutions cooperating in the field of research, determination of standards and the exchange of knowledge and good practices to improve the health of the young European generation;
- integrate, where possible, asthma and allergic diseases in children as a priority in current and future European research and action programmes and take into account the outcome of the reflection process into the implementation of the EU 2020 initiative.

Within the proposed Council Conclusion, a few manageable, achievable and cost-saving concrete actions should be prioritized. Two are specific to allergy and asthma and two are generic and in line with the UN Resolution 26 as proposed by the WHO Global Alliance against Chronic Respiratory Diseases (GARD) 4.

1. To disseminate and implement in all EU countries cost-saving national or regional asthma plans based on the Finnish and Polish experiences.
2. To sustain and expand a network of cooperation within the EU on allergy and asthma. It will include scientific and other activities related to public health with all stakeholders including patient's organizations. GA^2^LEN has already established such a network in almost all Member States to create referral centres for treating allergies and asthma. The network should be based on such domestic centres as elementary units regulating and implementing the relevant policy in individual countries, using the best practices, stemming from the international cooperation taking into account e-health and the potential contribution of other relevant policy areas.
3. To strengthen the fight against active and passive smoking in all EU countries at all levels.
4. To integrate chronic respiratory and allergic diseases in children in the NCD action plan to prevent and manage NCDs and improve healthy and active ageing.

## Abbreviations

ARIA: Allergic Rhinitis and its Impact on Asthma
COPD: chronic obstructive pulmonary diseases
CRD: chronic respiratory disease
ECRHS: European Community Research Health Survey
EFA: European Federation of Allergy and Airway Diseases Patients Association
FAST: Therapies for Food Allergy
FP: Framework Programme (European Union)
GA^2^LEN: Global Allergy and Asthma European Network (FP6)
GINA: Global Initiative for Asthma
ISAAC: International Study of Allergy and Asthma in Children
MeDALL: Mechanisms of the Development of Allergy (FP7)
NCD: noncommunicable disease
PreDicta: Postinfectious immune reprogramming and its association with persistence and chronicity of respiratory allergic diseases
